# Urinary Bladder Perforation Due to Foley Catheter: A Case Report and Review of Literature

**DOI:** 10.7759/cureus.32887

**Published:** 2022-12-23

**Authors:** Abdulla Nidal, Javereeya Abdul Jabbar, Yousif Habib Al Abboudi

**Affiliations:** 1 Department of Surgery, Dubai Health Authority, Dubai, ARE

**Keywords:** iatrogenic complication, surgery, catheter, exploratory laparotomy, perforation, bladder

## Abstract

Urinary bladder perforation is one of the few surgical emergencies prone to misdiagnosis, leading to a high mortality rate. Our case highlights patient management in such cases and reviews similar reports to increase awareness about patients with indwelling catheters and suspicion of bladder perforation.

A 73-year-old patient with decompensated heart failure developed severe abdominal pain, abdominal distention, and hematuria following a Foley catheter insertion. Computed tomography (CT) raised suspicion of bladder perforation. Exploratory laparotomy revealed serosanguinous fluid in the abdomen and rupture of the bladder dome. Bladder repair was performed, and the patient was monitored post-operatively under intensive care, with an uneventful recovery before discharge.

There are many causes of bladder perforation. It occurs most commonly due to traumatic incidence, iatrogenic instrumentation, or spontaneous rupture. It is a rare complication, accounting for 0.002% of all hospital admissions. According to the Centers for Disease Control (CDC), 12-15% of patients receive a urinary catheter during their hospital stay; therefore, it is important to consider complications of catheterization and their management.

Bladder rupture can present with non-specific symptoms leading to delayed management. We recommend clinical attention to patients with urinary catheter insertion presenting with severe abdominal pain, difficulty voiding, or hematuria to rule out the possibility of perforation. Rapid diagnosis and accurate treatment of such cases are crucial for an uneventful recovery.

## Introduction

Urinary bladder perforation is considered to be a relatively rare complication. It can occur due to abdominal or pelvic trauma, but it may also be an iatrogenic complication such as post-surgical procedures or may be spontaneous in nature [1].

An unusual cause of bladder rupture is an indwelling Foley catheter. It is a serious complication accompanied by high mortality and requires accurate diagnosis and prompt management. The mortality and morbidity rate associated with this condition is approximately 50%. Most of it is attributed to late diagnosis due to non-specific symptoms leading to delay in management [2].

Here we describe a case of a 73-year-old male with multiple comorbidities, who was initially admitted to the cardiology unit due to progressive shortness of breath. The patient was catheterized as he had developed acute-on-chronic kidney failure with decreased urine output. Two days after insertion of the indwelling catheter, the patient developed severe abdominal pain, distention, and hematuria. Abdominal computed tomography (CT) was performed, which showed intraperitoneal fluid collection with suspected bladder rupture.

## Case presentation

The 73-year-old male is a known case of heart failure with preserved ejection fraction, chronic atrial fibrillation, severe aortic stenosis with moderate-to-severe mitral regurgitation, adenocarcinoma of the colon, stage 4 chronic kidney disease, multiple strokes with a history of intracerebral brain hemorrhage with left-sided hemiparesis, chronic hypertension, hypothyroidism, and diabetes mellitus.

The patient developed hypertension and hypothyroidism in 2010 for which he is on amlodipine, furosemide, hydralazine, and levothyroxine. During the same year, he was diagnosed with colon cancer and underwent a right hemicolectomy. Histopathology confirmed moderately differentiated adenocarcinoma stage pT4 pN1 pMx, Dukes stage 1. After surgical management, the patient underwent eight cycles of adjuvant chemotherapy "Xelox" (capecitabine, oxaliplatin). Furthermore, the patient was diagnosed with diabetes in 2018 and managed with insulin and linagliptin. He developed stage 4 chronic kidney disease without dialysis in 2021, followed by heart failure with normal ejection fracture (55-60%) in 2022, and has a chronic history of atrial fibrillation and is on apixaban. The patient underwent transcatheter aortic valve replacement in 2022 due to severe, symptomatic aortic stenosis. He is also a known case of seizures, and is on levetiracetam.

He was bedridden since 2021 due to an L3 vertebral fracture and is on conservative management from the orthopedics team. He has had multiple admissions under cardiology and presented this time with similar complaints, which include progressive shortness of breath for five days, associated with orthopnea and paroxysmal nocturnal dyspnea, and bilateral lower limb swelling. He did not have any cough, palpitations, chest pain, or fever. He was admitted as a case of acute decompensated heart failure and pulmonary edema with suspicion of chest infection; hence, he was admitted to the cardiac care unit (CCU) and was under non-invasive ventilation.

Treatment for his condition was initiated per protocol including furosemide and spironolactone where he initially responded and passed a good amount of urine (1,500 mL per 24 hours) with a net negative 1,329 mL per 24 hours. However, over the next few days, his kidney function began to worsen, with an increased creatinine level of 5.5 mg/dL from a baseline of creatinine of ~3.2 mg/dL. The nephrology team identified an acute on top of chronic kidney disease that ultimately led to low urine output, and hence a urinary catheter was placed to measure urine output.

Two days after inserting the catheter, the patient developed sudden severe abdominal pain, abdominal distention, and hematuria. The patient also opened the bowel and passed a large amount of stool. However, no fever or vomiting was reported and the patient was vitally stable. During this time, there was a progressive rise in white blood cell count, increasing to 14.5x10^9^/L from a baseline of 7.2x10^9^/L, along with a decrease in hemoglobin level to 8.9 g/dL from 11.0 g/dL. His enoxaparin medication was discontinued.

**Figure 1:**
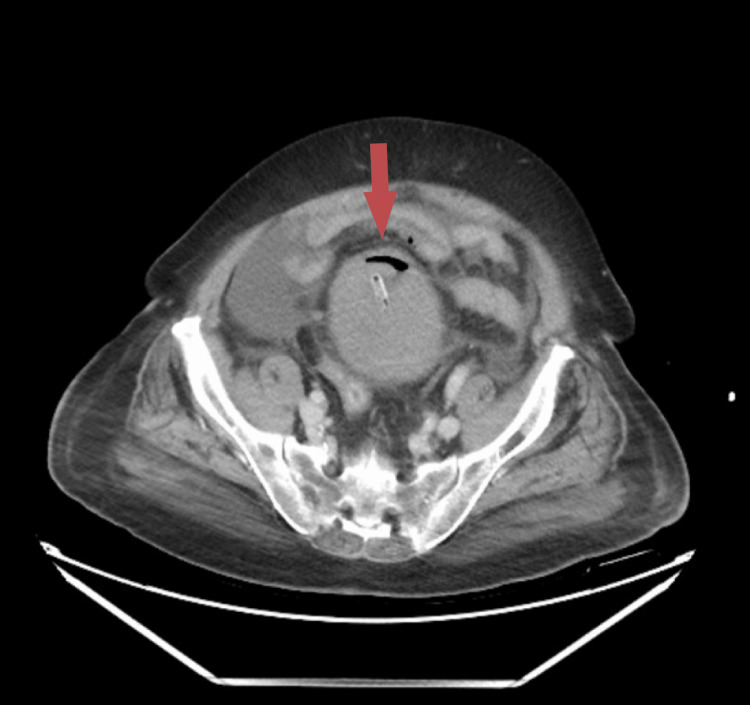
CT of the kidney, ureter, and bladder (axial view) showing emphysematous cystitis complicated with bladder rupture and large high density intra-abdominal collection

**Figure 2:**
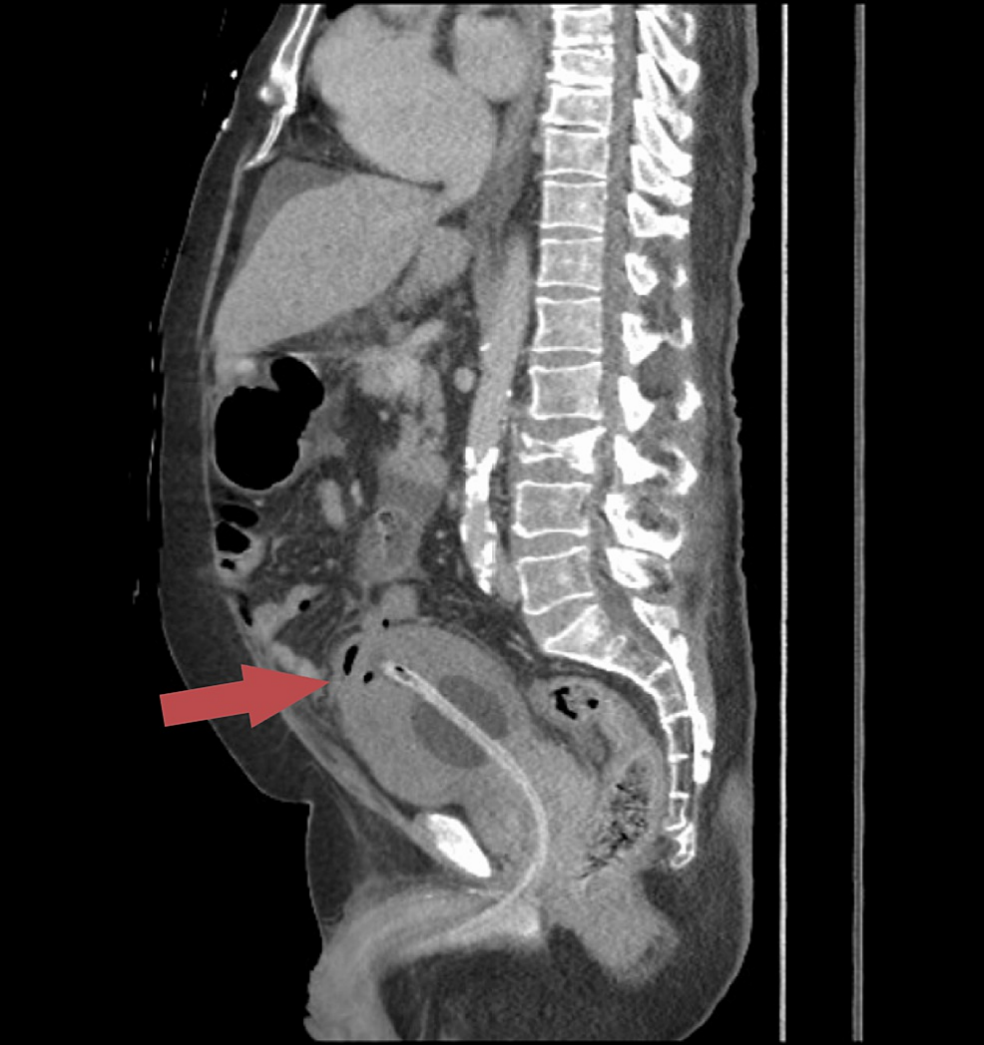
CT of the kidney, ureter, and bladder (sagittal view) showing emphysematous cystitis complicated with bladder rupture and large high density intra-abdominal collection

Our general surgery team was consulted due to the sudden onset. On examination, the patient had a distended abdomen with generalized tenderness and voluntary guarding despite a soft non-rigid abdomen. We, then, performed a CT of the kidney, ureter, and bladder, which showed evidence of emphysematous cystitis complicated with bladder rupture and extensive, high-density intra-abdominal collection. A large prostatomegaly showing irregular cystic areas with peripheral enhancement within the parenchyma was also noted, likely suggestive of abscess formation (Figures 1, 2). The urology team was immediately consulted, and a three-way catheter was inserted due to frank hematuria post-catheter.

**Figure 3:**
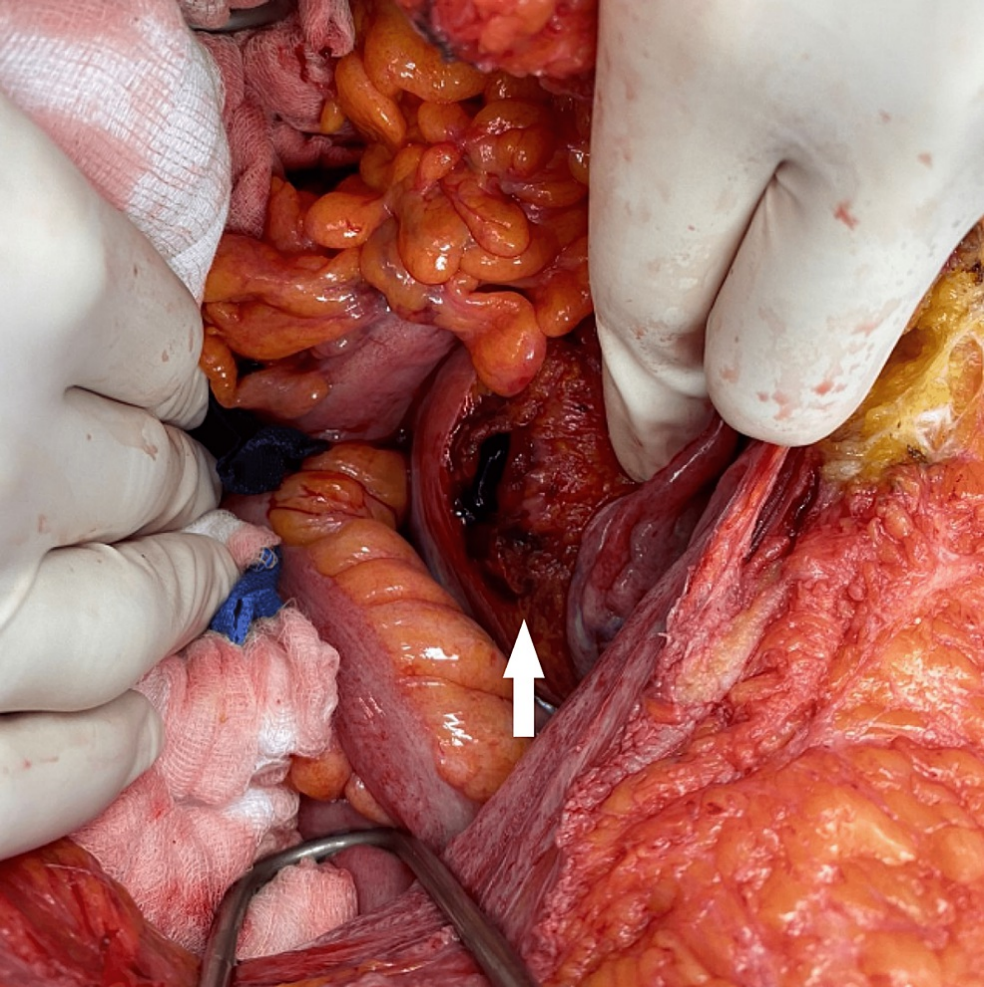
Intra-operative picture showing bladder dome perforation

CT revealed peritoneal cavity fluid collection with suspected bladder rupture, and thus a decision was made to take the patient for urgent exploratory laparotomy. During the surgery, a rupture of the bladder dome was confirmed (Figure 3) with 1 L of serosanguinous fluid in the abdomen, adhesions between bowel loops, and the anterior abdominal wall. The patient was taken for a joint operation with the urology and general surgery teams. A three-way catheter was inserted, and the balloon was inflated with 20 cc of normal saline. The bladder was irrigated and repaired with Vicryl 3/0 sutures with two layers of repair around the bladder dome at the site of the rupture. A 20-Fr drain was left in situ at the preperitoneal space. Abdominal washout was performed with drainage of the fluid and adhesiolysis. Post-operatively, he was admitted to the surgical ICU where he was kept intubated and sedated. Ventilatory support was weaned down as tolerated.

He was further monitored in the CCU. Over the days, the patient’s hematuria was improving along with inflammatory markers. Procalcitonin was down-trending from 4.79 ng/mL to 3.93 ng/mL, CRP from 297 mg/L to 277 mg/L, creatinine levels from 5.4 mg/dL to 3.2 mg/dL, and urea from 155 mg/dL to 95 mg/dL. The patient had an uneventful recovery and was discharged from general surgery care on April 2.

## Discussion

Bladder perforation is a relatively rare complication, with an incidence of 0.0007% and 0.002% of all hospital admissions [2]. This statistic is trivial especially relative to the number of patients receiving a urinary catheter during their stay at the hospital, which is reported to be 12-25% of the patients admitted according to the CDC (Centers for Disease Control) [2]. Therefore, it is vital to consider the potential complications of chronic or recurrent catheterization and the ways to manage them.

Gross hematuria, pelvic/lower abdominal pain, and difficulty voiding are the typical symptoms of urinary bladder perforation. However, patients may also present with urinary ascites, acute renal failure, and sepsis in cases of spontaneous bladder perforation. Patients reported having pelvic fractures should raise suspicion for all possible pelvic injuries including bladder perforation among others.

Diagnostics of bladder perforation have witnessed a shift from fluoroscopic cystography, which was traditionally used. Currently, the modality of choice is CT with contrast. This change came along due to the time-consuming nature of fluoroscopic cystography, which created the need to adopt a faster modality [1].

A case from the literature highlighted the importance of accurate diagnosis and urgent treatment in cases of bladder perforation as they are considered surgical emergencies. They reported the death of a 68-year-old female admitted to the ICU due to dyspnea, preceded by a full septic workup. She received a Foley catheter and was under high dependency care. The patient developed severe abdominal pain on the 10th day of admission associated with reduced urine output. Gross blood was seen in the urine after replacing the Foley catheter. Free peritoneal fluid in the abdomen, pelvis, and around the bladder, as well as left rectus sheath hematoma, was revealed in the CT abdomen and pelvis. CT cystogram confirmed the diagnosis and showed perforation at the bladder dome. Unfortunately, the patient developed hypovolemic shock due to significant blood loss, and despite vasopressor support, blood transfusion, and arranging transfer to a higher facility hospital, the patient died [2].

This parallels our patient's case with the demographics, presentation, and approach to diagnosis. The prompt decision to undergo a CT scan and urgent shift to the operation theater following the report further emphasized the vitality of efficient approach and decision-making.

Although very few cases of spontaneous bladder rupture have been reported in the literature, it is crucial to consider that the majority of cases with spontaneous rupture have an underlying cause. Underlying causes may be inflammatory, candida, or gonorrheal infections, as well as obstructed labor in pregnancy. Diabetics and alcoholics are at an increased risk of spontaneous bladder perforation due to altered bladder sensitivity. Decreased bladder sensitivity in diabetics predisposes them to urinary retention further leading to recurrent infections [3]. With the rich background of comorbidities, our patient was susceptible to perforation especially given the recurrent admissions and numerous catheter insertions for long durations.

It is important to note that patients enduring perforation are usually admitted for other health conditions unrelated to the perforation. Another example is that of a 67-year-old male, who's a known case of diabetes, hypertension, and dyslipidemia admitted due to left foot pain, swelling, and a pustular plantar ulcer on the foot. A urinary catheter was inserted, and the patient underwent debridement and management during his admission. He developed severe suprapubic abdominal pain, a guarded abdomen, fever, and vomiting on the third day of his hospital course. Upon exploration, a 3 x 3 cm perforation at the dome of the urinary bladder, inflamed mucosa, and collection of pus were noted [3].

Catheter-associated bladder injuries are usually considered to be one of the underlying causes of perforation, as opposed to being a direct cause. Patients who develop bladder perforation due to the Foley catheter insertion may present with atypical symptoms. An interesting report conducted in East Carolina University Brody School of Medicine/Vidant Medical Center described a case of a urinary bladder perforation in a 62-year-old African American female. Significant medical history included end-stage renal disease and oliguria; therefore, due to her distended bladder and sepsis workup, a Foley catheter was inserted. In the next 24 hours, the patient experienced abdominal discomfort and clinically deteriorated with findings of significant hypotension and tachycardia. Imaging confirmed bladder perforation, which was possible due to urinary catheter placement [4]. This is contrasted with our case where significant abdominal pain, abdominal distention, and hematuria were exhibited alongside oliguria.

Long-term catheterization can predispose patients to inflammatory changes within the bladder wall, which further promotes the occurrence of perforation and small bowel fistulation. The clinical course of a 65-year-old male who presented with abdominal distension decreased urine output for one day and nausea includes a medical history positive for prostate cancer, post-external beam radiation therapy 12 years ago, and an indwelling catheter for two years. The patient's urinary output decreased paradoxically right after urinary catheter placement. He did not show any signs of peritoneal inflammation; however, CT revealed that the catheter outside the bladder was forming an enterovesical fistula with an adjacent bowel loop, leading to obstruction. The patient underwent an emergent laparotomy. Acute ulcerative and transmural inflammation was found in the histopathology reports [5].

A large number of patients are kept with urinary catheters due to regular urinary output charting or for monitoring after surgical urethral or bladder repair. This can lead to various complications such as bladder stones, recurrent urinary tract infections, and iatrogenic hypospadias. Another case report highlighted an instance where the tip of the urinary catheter eroding through the bladder dome was seen on the CT of an 80-year-old male with a history of neurogenic bladder. Furthermore, post-0urgent laparotomy and adhesiolysis, a new open-tip catheter was inserted to prevent the recurrence of bladder erosion. However, it is important to understand the detrimental consequences of chronic catheterization and that such patients need regular and high levels of care [6].

Yet another case of a 6-mm perforation at the dome of the urinary bladder was discovered in an 86-year-old man in the setting of his own house as he would regularly get his catheter exchanged by a home-visiting nurse. On presentation, he had lower abdominal tenderness with guarding, and cloudy urine in the drainage bag. On attempting to irrigate, there was no return, which is a vital factor in suspecting bladder rupture. After appropriate imaging, followed by exploratory laparotomy, it was found that the inflated balloon of the Foley catheter was floating in the peritoneal cavity. Complicated peritonitis is a major consequence of delay in surgical treatment in patients with urinary bladder perforation [7].

Stable patients with isolated bladder injury are the only candidates for laparoscopic repair according to Marchand et al. [8]. However, our patient was septic and unstable and, hence, required a life-saving explaratory laparotomy.

Usually, intraperitoneal urinary bladder perforations are treated and managed surgically with repair of the defect. In rare cases where the patient is not fit for surgery and is not in a life-threatening condition, non-operative approaches may be explored as reported by Zhan et al. and Okuda et al. [9,10].

## Conclusions

Urinary bladder perforation, a surgical emergency, is known to have high morbidity and mortality rates attributing to non-specific clinical symptoms and delayed diagnosis. Accurate diagnosis and prompt intervention in these cases are crucial to ensure good prognosis for such patients. Our case is a successful example of how a rapid diagnosis was made and managed. Furthermore, our report adds a review of multiple cases with similar presentations and various treatment options in order to increase awareness about patients with indwelling catheters and suspicion of bladder perforation.
